# A novel resveratrol derivative induces mitotic arrest, centrosome fragmentation and cancer cell death by inhibiting γ-tubulin

**DOI:** 10.1186/s13008-019-0046-8

**Published:** 2019-04-10

**Authors:** Gianandrea Traversi, David Sasah Staid, Mario Fiore, Zulema Percario, Daniela Trisciuoglio, Roberto Antonioletti, Veronica Morea, Francesca Degrassi, Renata Cozzi

**Affiliations:** 1 Department of Science, University of “Roma Tre”, Rome, Italy; 2 Department of Biochemical Sciences ‘A. Rossi Fanelli’, Sapienza University of Rome, Rome, Italy; 3 Institute of Molecular Biology and Pathology, National Research Council of Italy (CNR), c/o Sapienza University of Rome, Rome, Italy; 4 Preclinical Models and New Therapeutic Agents Unit, IRCCS-Regina Elena National Cancer Institute, Rome, Italy

**Keywords:** Resveratrol analogues, Tubulin polymerization, Cancer cell proliferation, Centrosome fragmentation, γ-tubulin

## Abstract

**Background:**

Resveratrol and its natural stilbene-containing derivatives have been extensively investigated as potential chemotherapeutic agents. The synthetic manipulation of the stilbene scaffold has led to the generation of new analogues with improved anticancer activity and better bioavailability. In the present study we investigated the anticancer activity of a novel trimethoxystilbene derivative (3,4,4′-trimethoxylstilbene), where two methoxyl groups are adjacent on the benzene ring (ortho configuration), and compared its activity to 3,5,4′-trimethoxylstilbene, whose methoxyl groups are in meta configuration.

**Results:**

We provide evidence that the presence of the two methoxyl groups in ortho configuration renders 3,4,4′-trimethoxystilbene more efficient than the meta isomer in inhibiting cell proliferation and producing apoptotic death in colorectal cancer cells. Confocal microscopy of α- and γ-tubulin staining shows that the novel compound strongly depolymerizes the mitotic spindle and produces fragmentation of the pericentrosomal material. Computer assisted docking studies indicate that both molecules potentially interact with γ-tubulin, and that 3,4,4′-trimethoxystilbene is likely to establish stronger interactions with the protein.

**Conclusions:**

These findings demonstrate the ortho configuration confers higher specificity for γ-tubulin with respect to α-tubulin on 3,4,4′ trimethoxystilbene, allowing it to be defined as a new γ-tubulin inhibitor. A strong interaction with γ-tubulin might be a defining feature of molecules with high anticancer activity, as shown for the 3,4,4′ isomer.

**Electronic supplementary material:**

The online version of this article (10.1186/s13008-019-0046-8) contains supplementary material, which is available to authorized users.

## Background

Resveratrol (RSV) is one of the most intensively studied natural compounds, due to its anticarcinogenic activity in many cancer cell lines and animal studies [1]. However, the efficacy of RSV beneficial effects in human cancer is still an open question [2–4]. In this respect, RSV poor bioavailability represents a limitation for it use. To overcome this problem and ameliorate metabolism, different modifications have been introduced in the stilbene scaffold. As an example, methoxy derivatives, where two or three RSV hydroxyl groups are substituted with methoxyl ones, show increased lipophilicity, resulting in better bioavailability and higher antioxidant properties [5, 6]. We have recently shown that 3,5,4′-trimethoxystilbene (3,5,4′-TMS), whose stilbene scaffold is linked to three methoxyl groups, alters microtubule (MT) polymerization dynamics in cancer cells. Specifically, 3,5,4′-TMS induced multipolar spindles and mitotic arrest coupled to reduced cell proliferation and increased apoptotic death through mitotic catastrophe. On the contrary, the di-methoxy derivative pterostilbene was found not to affect MT dynamics [7].

MTs, consisting of α/β-tubulin heterodimers, play a pivotal role in mitosis by creating the mitotic spindle. In most somatic cells, MT nucleation occurs from the centrosome, which consists of a pair of centrioles surrounded by pericentrosomal material. This pericentrosomal material comprises also γ-tubulin, a homologue of αβ-tubulin, which acts as a nucleating agent by associating into a ring complex from where αβ-tubulin dimers assemble to polymerize into MTs [8]. Literature data show that MT dynamics is altered in cancer cells [9–11] and MT targeting agents are of great interest to inhibit mitotic division of these cells. These molecules, known as antimitotic drugs, bind different domains within MTs (e.g., laumalide, taxane, colchicine and vinca alkaloid sites) and affect MT stability [12, 13]. Computational docking studies suggested that 3,5,4′-TMS interacts with the colchicine-binding hydrophobic pocket of β-tubulin [14]. This finding is in line with our observation that 3,5,4′-TMS inhibits MT polymerization and, thereby, produces spindle multipolarity, DNA hypodiploidy and multinucleation in cancer cells characterized by supernumerary centrosomes [7].

**Fig. 1:**
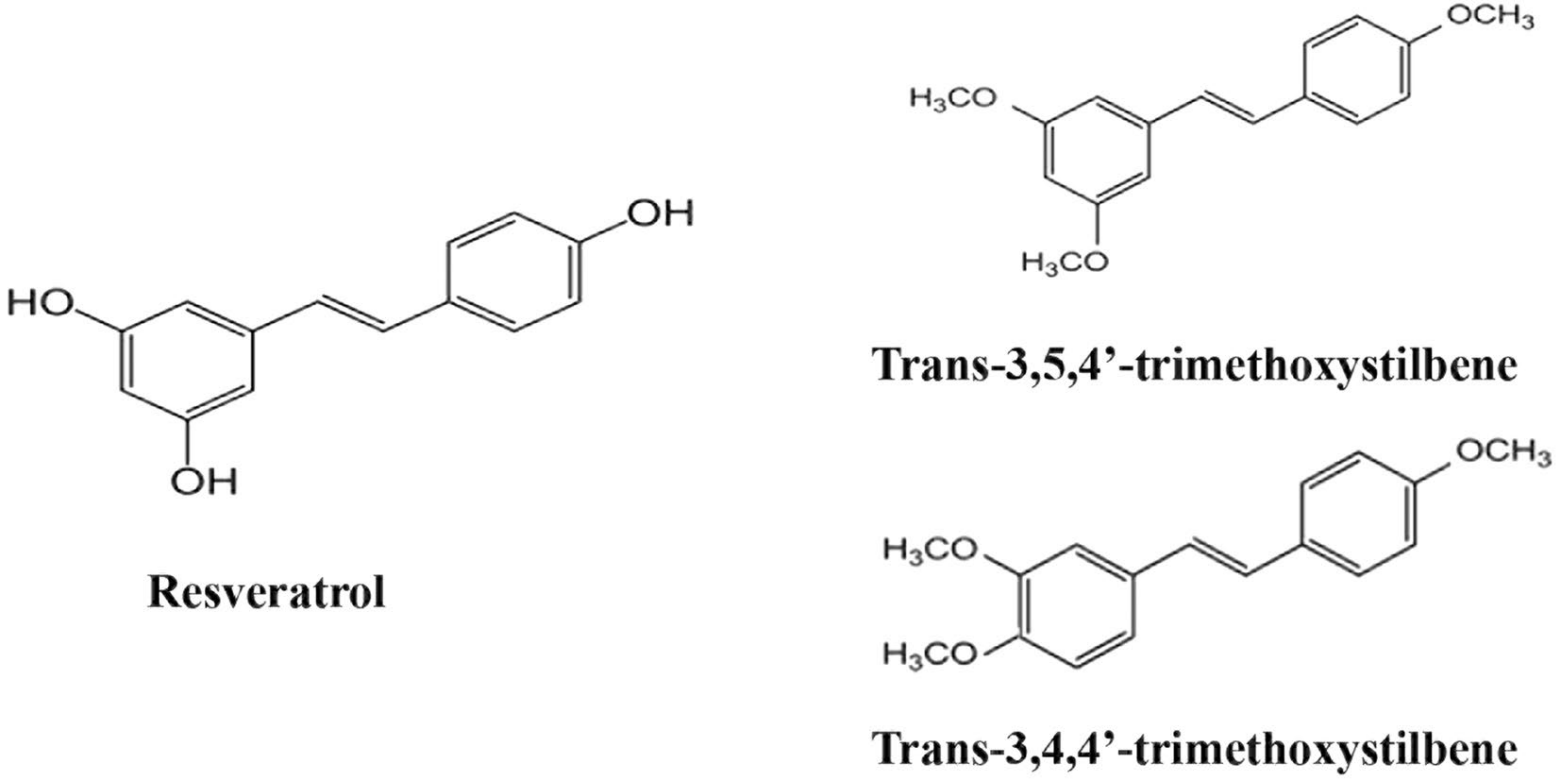
Chemical structure of resveratrol, the trimethoxy- resveratrol derivative 3,5,4′-trimethoxystilbene (3,5,4′-TMS) and its analogue 3,4,4′-trimethoxystilbene (3,4,4′-TMS)

Research on new trimethoxy-substituted RSV analogues is important to identify the structural determinants responsible for inhibiting cancer cell proliferation. In the present study, we have investigated the anti-cancer activity of 3,4,4′-trimethoxystilbene (3,4,4′-TMS), a novel trimethoxy stilbene derivative that differs from 3,5,4′-TMS for the presence of two methoxyl moieties on adjacent positions of the benzene ring (*ortho* configuration) as opposed to the *meta* configuration of 3,5,4′-TMS (Fig. 1) and compared the biological effects of these compounds. Computational docking studies have also been performed to investigate the possibility and mode of interaction of these molecules with tubulins.

Our results provide evidence that the presence of the two methoxyl groups in the *ortho* configuration renders 3,4,4′-TMS more efficient than the 3,5,4′ isomer in producing cell death in colorectal cancer cells, possibly through a stronger interaction with γ-tubulin.

## Results

### 3,4,4′-TMS is more effective than 3,5,4′-TMS in determining growth inhibition and mitotic arrest in cancer cells

**Fig. 2:**
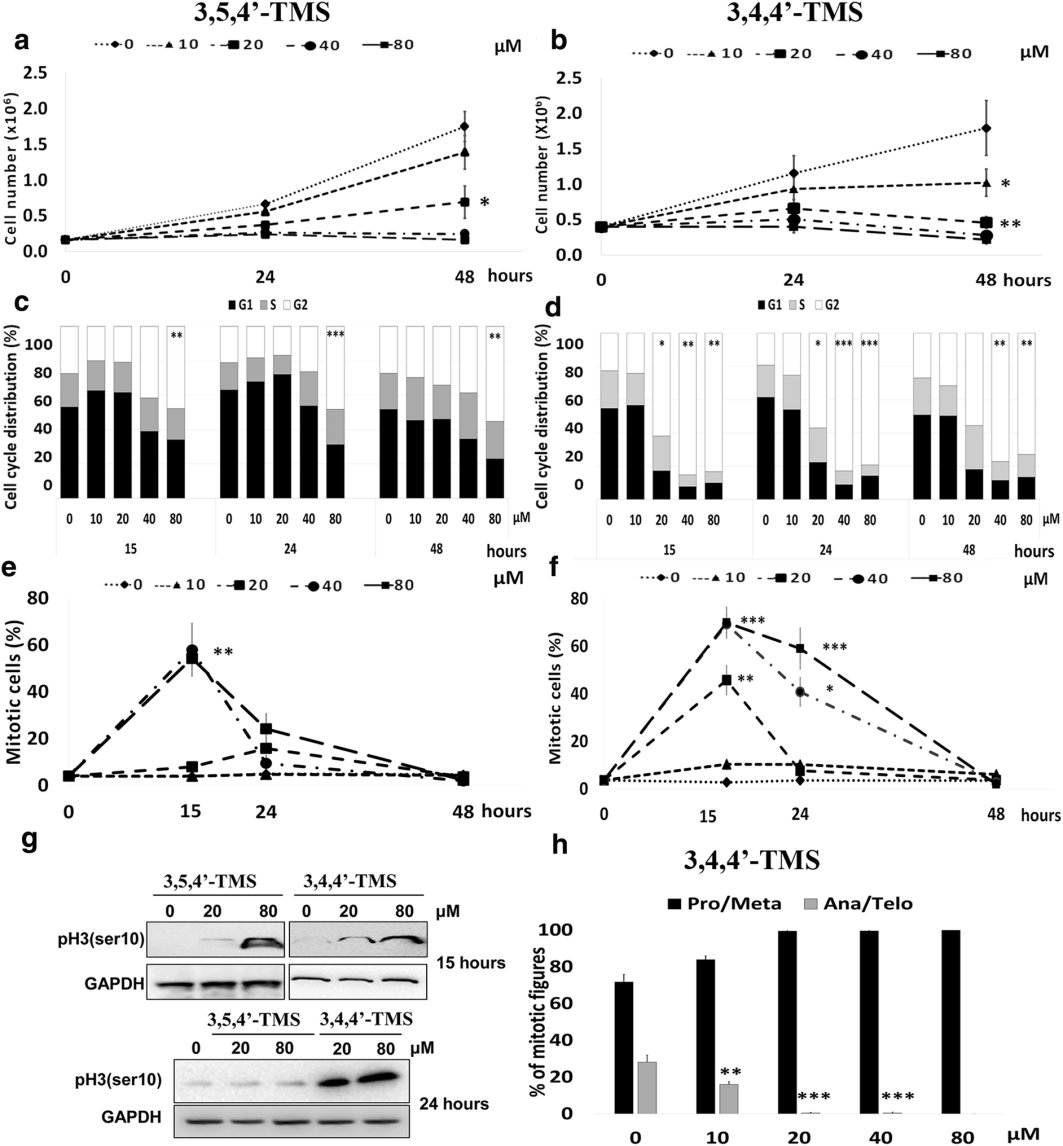
Inhibition of cell growth and mitotic division after exposure to 3,5,4′-TMS or 3,4,4′-TMS. Growth curves of HCT116 cells treated with different concentrations of 3,5,4′-TMS (**a**) or 3,4,4′-TMS (**b**). Time course analysis of cell cycle distribution by flow cytometry following 3,5,4′-TMS (**c**) or 3,4,4′-TMS (**d**) exposure. Frequency of mitoses following 3,5,4′-TMS (**e**) or 3,4,4′-TMS (**f**) exposure. **g** Western blot analysis of ser10 phosphorylated histone H3 (pH3(ser10)) after 15 or 24 h exposure to 3,5,4′-TMS or 3,4,4′-TMS. GAPDH is used as loading control. **h** Frequency of prometa/metaphase cells (Pro/Meta) and anaphase/telophase cells (Ana/Telo) in HCT116 mitoses collected after 15 h treatment with 3,4,4′-TMS. Values are the mean ± SE of 3–4 independent experiments. *: p < 0.05; **: p < 0.01; ***: p < 0.001 compared with controls

The potency of the two RSV methoxylated analogues in inhibiting cancer cell proliferation was assessed by analyzing cell growth, cell cycle progression and mitotic index in treated HCT116 cancer cells (Fig. 2). Both molecules drastically inhibited cell growth at doses higher than 20 μM, markedly at 48 h. 3,4,4′ TMS was more efficient than 3,5,4′ TMS in reducing cell proliferation since growth inhibition was statistically significant already at 10 μM (Fig. 2a, b). To better understand the mechanism of action of the two molecules, we examined cell cycle progression by flow cytometry in time course experiments. 3,4,4′-TMS was extremely potent in accumulating cells in the G2/M phases and reducing the fraction of G1 and S phase cells. Indeed, G2/M accumulation by 3,4,4′-TMS was statistically significant from 15 h time point and 20 μM dose, while 3,5,4′-TMS significantly accumulated cells in G2/M only at 80 μM (Fig. 2c, d and Additional file 1: Fig. S1). To distinguish whether cells were delayed in G2 or blocked in mitosis, mitotic index was measured to identify a possible mitotic arrest. 3,4,4′-TMS significantly increased the frequency of mitoses at 15 h treatment with 20 μM, whereas increased mitotic index was observed only from 40 μM 3,5,4′-TMS at the same time point. At 24 h the mitotic fraction decreased in 3,5,4′-TMS treated cells approaching control values, whereas mitotic index remained high up to 48 h after 3,4,4′-TMS treatment (Fig. 2e, f). These observations demonstrate that 3,4,4′-TMS induces a mitotic arrest at lower doses and for a longer time. These findings were confirmed by the analysis of Ser10 phosphorylation on histone H3, a widely established marker of mitosis (Fig. 2g). When the different mitotic figures were scored in 3,4,4′-TMS treated cells, anaphase and telophase cells were reduced starting from 10 μM and disappeared from the mitotic population at higher doses, indicating a spindle assembly checkpoint-dependent prometaphase arrest (Fig. 2h).

**Fig. 3:**
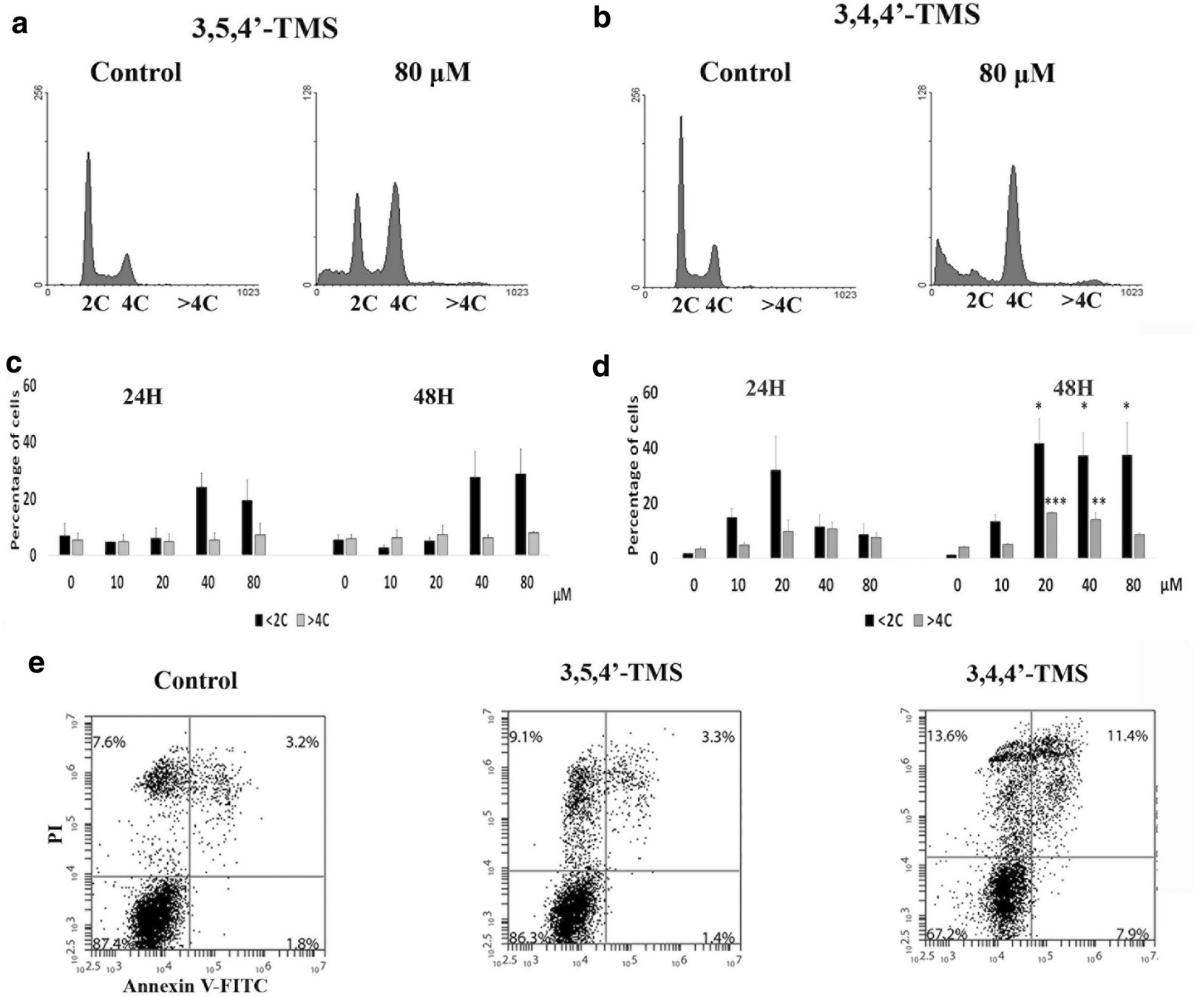
Induction of apoptotic cell death following exposure to 3,5,4′-TMS or 3,4,4′-TMS. Representative flow cytometric histograms of HCT116 cells at 48 h following 3,5,4′-TMS (**a**) or 3,4,4′-TMS (**b**) treatment. X axis = DNA content, Y axis = number of events. Quantitative analysis of the percentage of hypodiploid (< 2C) and polyploid (> 4C) cells after 24 or 48 h exposure to 3,5,4′-TMS (**c**) or 3,4,4′-TMS (**d**). Data are mean ± SE of 2–4 independent experiments. *: p < 0.05; **: p < 0.01; ***: p < 0.001 compared with controls. Flow cytometric analysis of Annexin V-FITC reactivity of HCT116 cells treated for 48 h with 20 μM of 3,5,4′-TMS or 3,4,4′-TMS (**e**)

Flow cytometry analysis of apoptosis (Fig. 3) showed that 3,4,4′-TMS significantly enhanced the frequency of hypodiploid cells at 48 h starting from the 20 μM dose (Fig. 3d). Under the same treatment conditions induction of apoptosis by 3,4,4′-TMS was also confirmed by annexin V staining (Fig. 3e). At 48 h treatment time mitotic index had returned to control levels (Fig. 2f), suggesting that treated cells undergo apoptosis when the mitotic arrest observed at 15 and 24 h is overcome. Finally, a minor fraction of cells exited mitosis as polyploid after 3,4,4′-TMS (Fig. 3d). Apoptosis from mitosis and exiting from mitosis as polyploid, are two outcomes of arrested mitosis. These cell fates indicate mitotic catastrophe [15] as the death pathway elicited by 3,4,4′-TMS.

### 3,4,4′ TMS and 3,5,4′-TMS differently affect mitotic MT organization

**Fig. 4:**
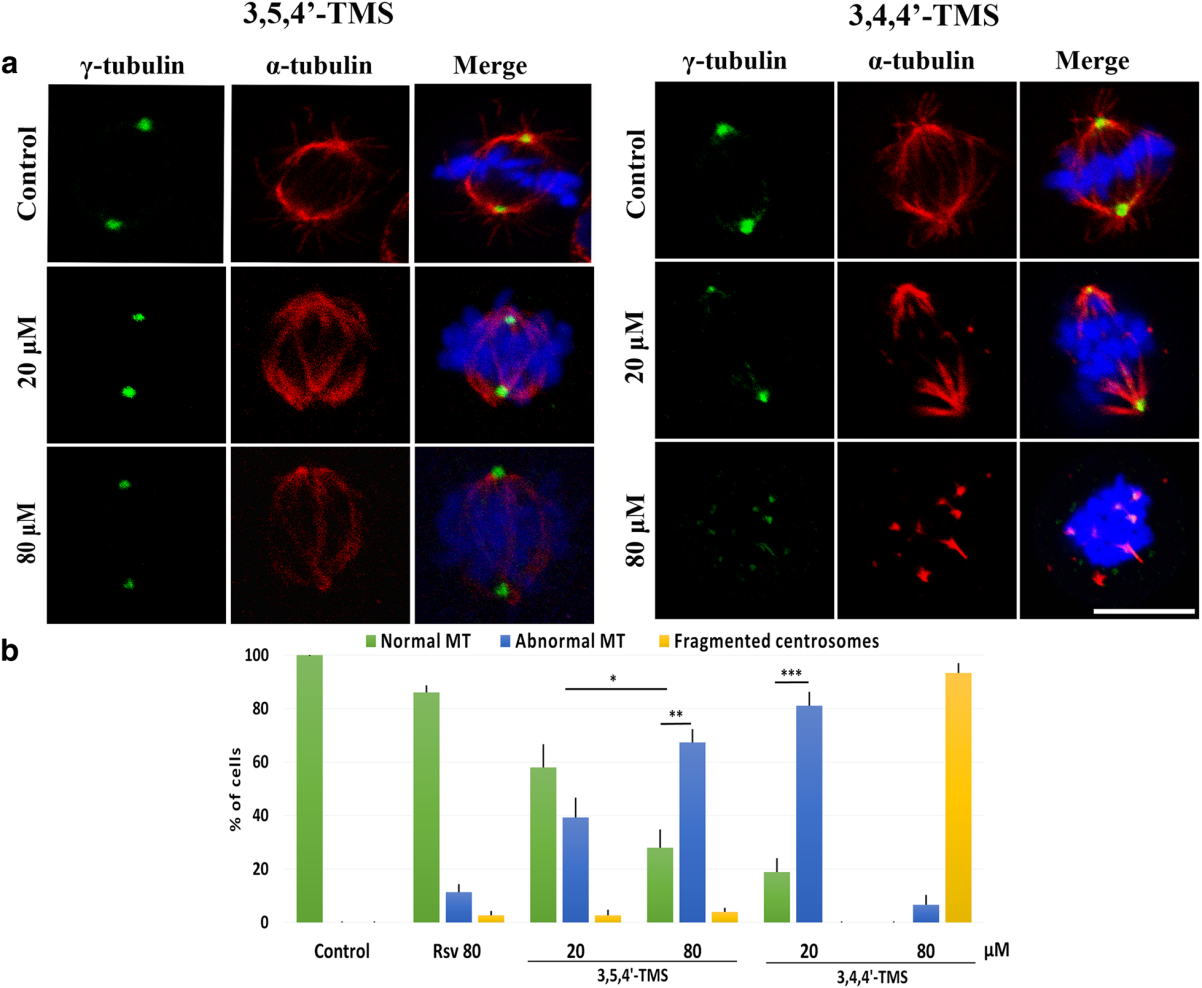
Alterations of mitotic spindle structure upon treatment with 3,5,4′-TMS or 3,4,4′-TMS. **a** HCT116 cells were treated with 20 or 80 µM 3,5,4′-TMS or 3,4,4′-TMS for 2 h, fixed and stained with anti-α-tubulin and anti-γ-tubulin antibodies. 3D projections of confocal images of untreated and treated cells are shown. Scale bar, 10 μm. **b** Quantitative analysis of MT and centrosome alterations in control and treated cells. Values are the mean ± SE of 2 independent experiments. *: p < 0.05; **: p < 0.01; ***: p < 0.001

**Fig. 5:**
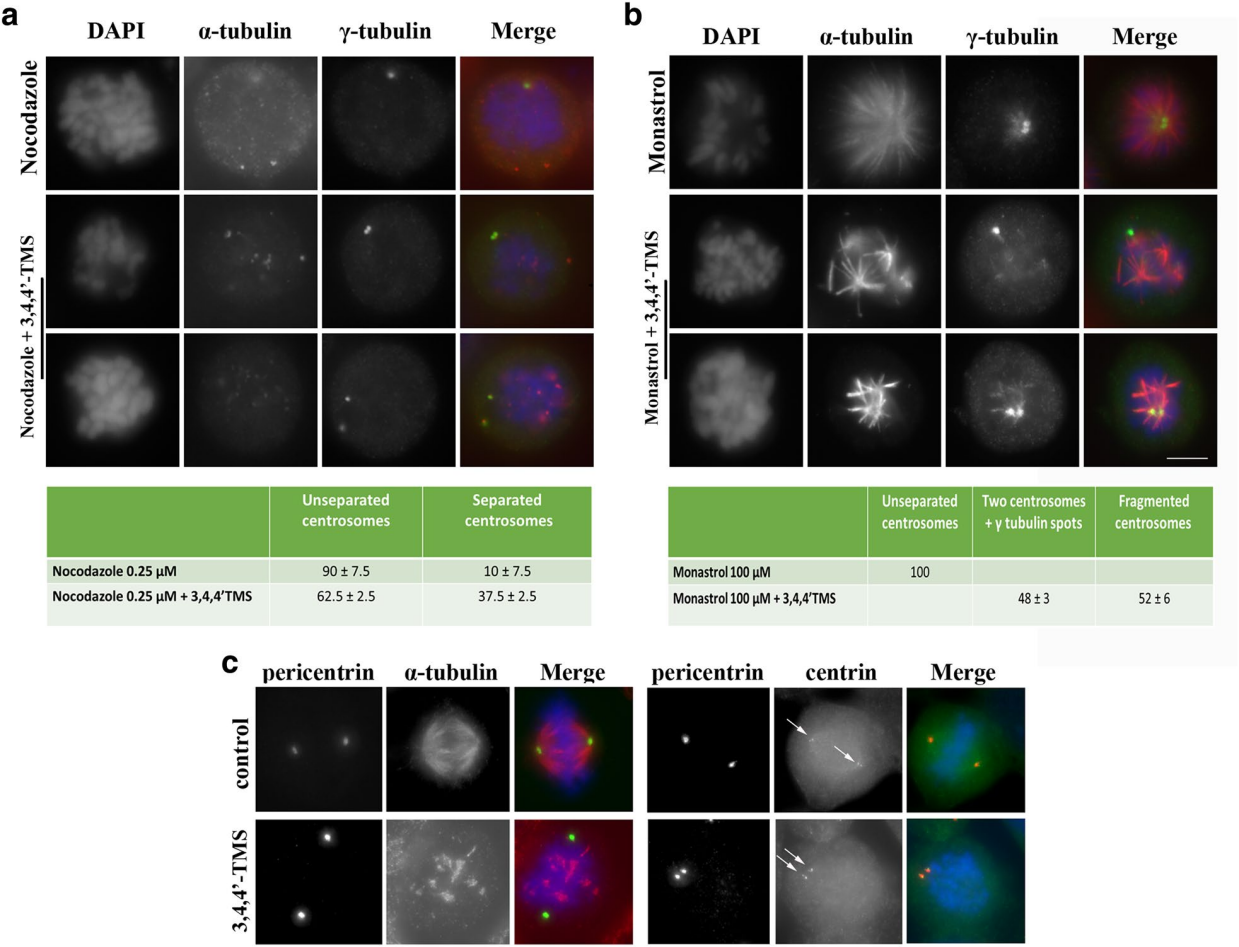
Centrosome fragmentation by 3,4,4′-TMS occurs during the early stages of mitosis. **a** HCT116 cells were treated for 2 h with 0.25 μM nocodazole or co-treated with nocodazole and 40 μM 3,4,4′-TMS. **b** HCT116 cells were treated for 2 h with 100 μM monastrol or co-treated with monastrol and 40 μM 3,4,4′-TMS. In both cases cells were then immunostained with anti-α- and anti-γ-tubulin antibodies and DNA was counterstained by DAPI staining. The tables below report the percentage of cells with MT and centrosome alterations for the different treatments. Values are the mean ± SE of 2 independent experiments. **c** HCT116 cells were treated for 2 h with 80 μM 3,4,4′-TMS and then immunostained with anti-α- tubulin, pericentrin or centrin antibodies. The arrows point to centrin signals. Scale bar, 10 μm

Mitotic catastrophe is the death pathway associated with MT-disrupting agents. Since our previous work demonstrated that 3,5,4′-TMS acts as a tubulin depolymerizing agent in cancer cells, we visualized the MT-based mitotic spindle structure in 3,5,4′-TMS and 3,4,4′-TMS treated cells by confocal microscopy. In agreement with previous results, 3,5,4′-TMS treated mitoses showed disorganized MT arrays that did not interact with kinetochores, as also observed for the lower concentration of 3,4,4′-TMS (Fig. 4a). Strikingly, multiple spots of co-localizing α-tubulin and γ-tubulin signals were observed in > 80% of the mitoses when 3,4,4′-TMS was supplied at 80 μM in both diploid HCT116 (Fig. 4a, b) and chromosomally unstable SW620 colon cancer cells (Additional file 2: Fig. S2A). In SW620 cells 3,4,4′-TMS was also more potent than 3,5,4′-TMS in inducing apoptosis (Additional file 2: Fig. S2B and C), suggesting that 3,4,4′-TMS may inhibit cell proliferation and produce centrosome fragmentation and spindle multipolarity in multiple cancer cells. We ruled out centrosome amplification as the cause of multiple γ-tubulin signals, since no centrosome replication could have occurred in the short treatment time (2 h) applied in these experiments. Spindle multipolarity without centrosome amplification may derive from exacerbated spindle forces and/or defective centrosome integrity [16]. To search for molecular players involved in these processes, we immunostained cells for spindle pole associated proteins implicated in the assembly and maintenance of the mitotic spindle (Aurora A, TPX2, Kif2a), and found that these proteins correctly localized at spindle poles in both untreated and 3,4,4′-TMS treated cells (Additional file 3: Fig. S3). We then tested whether an unbalance of forces, generating an excessive pulling force on centrosomes was at the origin of centrosome fragmentation in 3,4,4′-TMS treated cells. To this aim, we depolymerized spindle MTs by adding the MT inhibitor nocodazole concomitantly with the RSV analogue and monitored centrosome fragmentation (Fig. 5a). Centrosomes remained intact when the two chemicals were supplied together, implicating that MT forces have a role in spindle pole fragmentation Interestingly, centrosomes appeared to be separated in a consistent fraction of cells (37.5%), suggesting that centrosome structure was weaker after 3,4,4′-TMS treatment. To identify the mitotic stage when fragmentation intervened, we used monastrol, an inhibitor of the Eg5 kinesin that is responsible for centrosome separation at M phase entry [17]. In the presence of the drug mitotic spindles were monopolar, due to polymerization of functional MTs without centrosome separation. When 3,4,4′-TMS was supplied together with monastrol, centrosomes fragmentation was maintained. Notably, in > 50% of cells at least one centrosome was completely fragmented (Fig. 5b). These observations indicate that fragmentation intervened before centrosome separation, due to an intrinsic structural weakness of the centrosomes. To investigate this point, we immunostained 3,4,4′-TMS treated cells for internal components of the centrosome, i.e. pericentrin and centrin, and found that they were unaffected (Fig. 5c). These findings demonstrated that fragmentation occurred at the external layer of the centrosome, i.e. at the pericentrosomal material, and suggested that γ-tubulin may be the 3,4,4′-TMS target molecule since this protein is the main component of the pericentrosomal material.

### Computational studies of the interaction of 3,4,4′-TMS with γ-tubulin

**Fig. 6:**
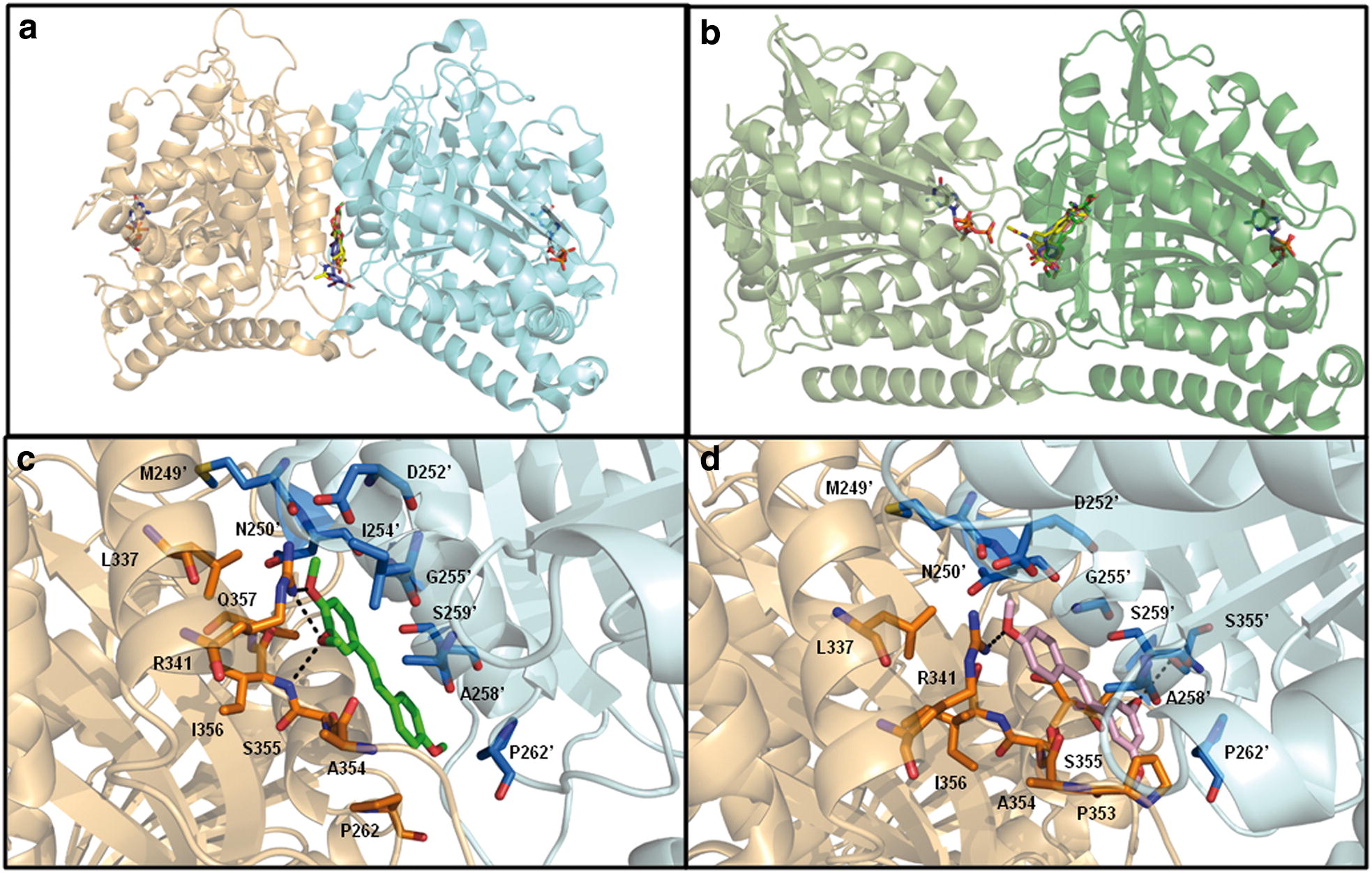
Interaction of 3,4,4′-TMS and related compounds with γ/γ- and α/β-tubulin dimers. **a** Predicted mode of interaction of 3,4,4′-TMS, 3,5,4′-TMS, combretastatin A4 and colchicine with γ/γ-tubulin dimers. The two γ-tubulin monomers are shown as ribbon and coloured gold and cyan, respectively. Both the GDP present in the structure and the docked ligands are shown as sticks and coloured by atom type: N, blue; O, red; P, orange; C, green, salmon, blue, yellow and grey in 3,4,4′-TMS, 3,5,4′-TMS, combretastatin A4, colchicine and GDP, respectively. **b** Predicted mode of interaction of 3,4,4′-TMS, 3,5,4′-TMS, combretastatin A4 and colchicine with α/β-tubulin dimers. The α- and β-tubulin monomers are shown as ribbon and coloured light and dark green, respectively. Both the GTP and GDP inherited from the template and bound to α- or β-tubulin monomers, respectively, and the docked ligands are shown as sticks and coloured as in **a**. **c** Close view of the interactions between 3,4,4′-TMS and γ-tubulin. 3,4,4′-TMS is coloured as in **a**. γ-tubulin residues having at least one atom within 4.0 Å from 3,4,4′-TMS are labelled (residues belonging to the two monomers are differentiated by the absence and presence of an apex, respectively), shown as sticks and coloured by atom-type: N, blue; O, red; C, orange and light blue for the γ-tubulin monomer on the left and on the right, respectively. Hydrogen bonds are indicated by dashed lines. **d** Close view of the interactions between 3,5,4′-TMS and γ-tubulin. 3,5,4′-TMS is coloured as in **a**. γ-tubulin residues having at least one atom within 4.0 Å from 3,5,4′-TMS are shown as in **c**. Hydrogen bonds are indicated by dashed lines

To investigate the potential ability of 3,4,4′-TMS to interact with γ-tubulin, we performed docking simulations with the two RSV analogues 3,4,4′-TMS and 3,5,4′-TMS, and the two well-known α/β-tubulin inhibitors combretastatin A4 and colchicine, which have also been reported to interact with γ/γ-tubulin [18]. Both 3,4,4′-TMS and 3,5,4′-TMS, as well as combretastatin A4 and colchicine, are predicted to interact with both γ/γ and α/β-tubulin dimers by computational docking studies. For all the molecules, interaction occurs at largely overlapping binding sites, located at the inter-monomer interfaces (Fig. 6a, b), albeit with different affinities (Table 1). Additionally, the aforementioned ligands bind to a γ/γ-tubulin region partially overlapping to the site where the same ligands bind on α/β-tubulin, and to the colchicine binding site observed in the experimentally determined 3D structure of the complex with α/β-tubulin [19] (Fig. 6a, b and Additional file 4: Fig. S4 and Additional file 5: Fig. S5). Due to the different architecture of γ/γ- (“head-to-head”) and α/β-tubulin (“tail-to-head”) interfaces (compare GTP/GDP positions in Fig. 6a, b), only the γ-tubulin monomer structurally equivalent to β-tubulin can contribute homologous residues to the binding site, the other γ-tubulin monomer having a completely different orientation from that of α-tubulin in the α/β dimer.

**Table 1:** Autodock scores and affinity values for the interaction between 3,4,4′-TMS, 3,5,4′-TMS, combretastatin A4 or colchicine and γ/γ-tubulin (γ/γ) or α/β-tubulin (α/β) dimers

	ΔG (γ/γ)	Kd (γ/γ)	ΔG (α/β)	Kd (α/β)	ΔG (α/β)/ ΔG (γ/γ)	Kd (α/β)/ Kd (γ/γ)
3,4,4′-TMS	− 7.36	4.023	− 6.35	22.128	0.863	5.501
3,5,4′-TMS	− 7.02	7.141	− 6.37	21.393	0.907	2.996
Combretastatin A4	− 6.07	35.498	− 6.72	11.849	1.107	0.334
Colchicine	− 7.27	4.683	− 9.91	0.054	1.363	0.012

ΔG: best score calculated by Autodock for the most populated clusters. Kd (μM): predicted dissociation constant values calculated using the formula: Kd = exp(ΔG/(R*T))

Based on Kd values calculated from AutoDock scores (see Table 1), both colchicine and combretastatin A4 bind α/β-tubulin with higher affinity than γ/γ-tubulin (87 and threefold, respectively); conversely, both 3,4,4′-TMS and 3,5,4′-TMS have lower Kd values for γ/γ-tubulin (in the low μM range) than α/β-tubulin (in the tens of μM range). In particular, 3,5,4′-TMS and 3,4,4′-TMS affinity for γ/γ-tubulin is 3- and 5.5-fold higher than that towards α/β-tubulin. Analysis of the best energy models of γ/γ-tubulin in complex with 3,4,4′-TMS and 3,5,4′-TMS indicates that the former compound establishes three hydrogen bonds with the protein, involving O3 and O4 of the ligand and the side-chain NH1 group of R341, and O3 of the ligand and the main-chain N atom of I356 (Fig. 6c). The same hydrogen bond interactions cannot be established by 3,5,4′-TMS, due to the different position of O atoms (Fig. 6d). Indeed, with respect to 3,4,4′-TMS, the latter compound assumes a conformation rotated by about 180° around an axis perpendicular to the plane of the molecule and passing halfway through the *trans* double bond between C8 and C9. This conformation allows 3,5,4′-TMS to establish two hydrogen bonds only: one between the O4′ atom and NH1 of R341, the other between the O3 atom and the side-chain hydroxyl group of S355′. Thus, the analysis of the docking models supports the hypothesis of a higher affinity of γ/γ–tubulin for 3,4,4′-TMS with respect to 3,5,4′-TMS, and provides a molecular explanation for it.

## Discussion

The structural modification of natural products such as RSV offers opportunities for the rational design of new chemotherapeutic agents. Indeed, it has been demonstrated that the presence of three methoxy groups on the stilbene scaffold, while improving bioavailability and conferring higher antioxidant activity [6, 20], leads to increased antiproliferative activity and apoptotic death coupled to inhibition of tubulin polymerization [7, 21–24]. In this context we investigated the anticancer properties of two trimethoxy derivatives of RSV in human cancer cells. One molecule, 3,5,4′-TMS, was previously demonstrated to induce mitotic arrest through MT depolymerization [7]; the other one, 3,4,4′-TMS, was newly synthesized by our group to test the effect of a subtle modification, i.e. from meta to ortho configuration in the A-ring, on anti-cancer potency.

We assayed the two trimethoxy resveratrol derivatives for their ability to produce cell growth inhibition and apoptosis in HCT116 and SW620 cells. Here, we demonstrated that 3,4,4′-TMS is more efficient than 3,5,4′-TMS in reducing cell growth, in that it shows a strong antiproliferative effect at lower doses. This is associated with an accumulation of mitotic cells in prometaphase starting from 20 µM, a finding that identifies mitosis as the cellular process targeted by 3,4,4′-TMS. The compound significantly induces apoptotic death in HCT116 cells at 20 μM, as shown by the induction of hypodiploid and Annexin-V positive cells. At the same concentration, no significant apoptosis was observed after 3,5,4′-TMS. These findings demonstrate that the modification from meta to ortho configuration significantly enhances the anti-cancer activity of the chemical. Confocal analysis of mitotic cells in 3,4,4′-TMS treated cultures showed the presence of multiple small radial signals of α-tubulin with γ-tubulin at their centre. Combination studies using mitotic inhibitors demonstrated that these ectopic MT aggregates consisted of fragmented pericentrosomal material with aborted α-tubulin nucleation. On the contrary, 3,5,4′-TMS significantly impaired mitotic spindle assembly without producing centrosome fragmentation. These findings suggest that 3,4,4′ TMS might exert its action by interacting with γ-tubulin. This hypothesis is supported by molecular docking studies, which show that both 3,4,4′-TMS and 3,5,4′-TMS potentially interact with γ-tubulin. Interestingly, 3,4,4′-TMS is likely to have a better affinity with γ–tubulin because of its ability to establish three hydrogen bonds with the γ–tubulin dimer instead of the two formed by 3,5,4′-TMS.

The present study demonstrates that methylation of resveratrol leads to profound changes in the mode of action of the compound and highlights the relevance of the relative position of substituents for the specificity of the target molecule. The ortho configuration makes 3,4,4′-TMS more specific for γ-tubulin, as opposed to 3,5,4′-TMS, which has methoxyl substituents in meta configuration. Consequently, 3,4,4′-TMS may be considered as a new γ-tubulin inhibitor. Despite the importance of γ-tubulin for MT-dependent cellular functions, the identification of specific γ-tubulin inhibitors has lagged behind, due to the high homology of γ-tubulin with α-tubulin and the tiny amount of centrosome-associated tubulin in cells. Indeed, y-tubulin represents < 1% of the total tubulin content in the cell [25]. Recently, a fully validated γ-tubulin inhibitor has been reported in the literature, which was obtained through the chemical modification of known drugs interacting at the colchicine binding site in β-tubulin [26]. Using a similar approach, we have identified 3,4,4′ TMS as a potential γ-tubulin inhibitor.

Importantly, γ-tubulin has been found to be overexpressed in glioblastoma multiforme cancer [27, 28], as well as in non-small cell lung [29] and medulloblastoma [30] cells. Furthermore, centrosome amplification (closely linked to increased γ-tubulin cell content) is present in many cancer cells [31]. Beside promoting chromosome segregation defects and aneuploidy at mitosis [32, 33], increased MT nucleation capacity from centrosome amplification can increase cellular invasion [34], connecting centrosome amplification to advanced tumour stages and metastasis [35]. Reducing MT nucleation in cells showing supernumerary centrosomes through γ-tubulin inhibition may offer a novel route to reduce the aggressiveness of these tumour cells.

## Conclusions

Our study identifies γ-tubulin as a relevant target for inhibiting cancer cell proliferation. 3,4,4′ TMS or optimized derivatives of this molecule could represent promising therapeutic tools to treat very aggressive cancers, especially those characterized by centrosome amplification.

## Methods

### Chemicals

RSV, monastrol and nocodazole were purchased from Sigma-Aldrich (Saint Louis, MO). (E)-3,5,4′-TMS and (E)-3,4,4′-TMS were synthetized by classical olefin synthesis using Wittig reaction with a slight modification [36]. The ylide was generated by LiOH starting from the phosphonium salt. Then, the olefin products were obtained as mixture of Z and E isomers by reaction with benzaldehydes. The Z/E mixtures were converted to E-isomers by heating with catalytic amounts of iodine in refluxing heptane [37]. Molecular structures and details of the synthesis are reported in Additional file 7: Additional Information and Additional file 6: Fig. S6. Chemicals were dissolved in DMSO. DMSO concentration never exceeded 0.02% in the cultures.

### Cell cultures

HCT116 and SW620 colorectal cancer cells were maintained in Dulbecco’s Modified Eagle Medium (DMEM) High Glucose supplemented with 10% fetal bovine serum, 2% penicillin/streptomycin solution, 1% l-glutamine and 0.1% gentamicin, in a 37 °C humidified incubator with 5% CO_2_. All culture reagents were purchased from Euroclone (Milan, Italy).

### Cell proliferation assays

Cells were seeded in 25 cm^2^ flasks at a density of 4 × 10^5^ cells/flask 1 day before the experiment and then treated for 15, 24 or 48 h. At the end of treatment, an aliquot of each sample was collected to count the number of cells through a Z1 Counter (Beckman Coulter). The remaining cell suspension was centrifuged, incubated in a 3:1 distilled water/medium mixture for 5 min and fixed in a 3:1 methanol/acetic acid mixture. Finally, cells were dropped on slides and stained with the conventional Giemsa method. For each experimental point, 1000 cells were analysed to count the number of mitoses. At least 200 mitoses were analysed to identify the different mitotic figures.

### Analyses of cell cycle progression and apoptosis

Cells were seeded in 25 cm^2^ flasks at a density of 4 × 10^5^ cells/flask 1 day before the experiment and then treated for 24 or 48 h. At the end of treatment, cells were trypsinized, washed with PBS and fixed in a 1:1 cold methanol:PBS mixture. For cell cycle analysis, fixed cells were centrifuged, resuspended in a solution containing 50 μg/ml RNase A and 20 μg/ml propidium iodide. Cell death was analysed using the Annexin V-FITC apoptosis detection kit (eBioscience) on live cells. Flow cytometric analyses were carried out on an Epics XL apparatus (Beckman Coulter). Ten thousand events were collected from each sample and data were analyzed using WinMDI 2.9 software. For western blot analysis, 40 μg of total proteins were resolved in 4–12% gradient gels by SDS-PAGE. Nitrocellulose membranes were incubated with anti ser10 phospho H3 (Millipore) and with anti GAPDH (Santa Cruz) antibodies. Signals were revealed by enhanced chemoluminescence.

### Immunofluorescence microscopy and analysis

Cells were seeded on glass coverslips in 35 mm Petri dishes (2 × 10^5^ cells/dish) 2 days before the experiment and then treated for 2 h. At the end of the treatment cells were processed as described in [7]. Primary antibodies were anti-α-tubulin and anti-γ-tubulin antibodies (Sigma-Aldrich). Secondary antibodies were Alexa 488 anti-rabbit (Molecular Probes) and X-Red anti-mouse (Jackson Laboratories) antibodies. DNA was counterstained with fluorescent RedDot™2 dye (Biotium, Inc). Cells were viewed under a Leica TCS SP5 confocal microscope and processed with LAS AF V1.6.3 software (Leica Microsystems). Images shown are 3D projections of Z-stacks from ≈ 20 confocal sections acquired at 0.5 µm intervals. To analyze centrosomal proteins, cells were processed as described above. Primary antibodies were anti-KIF2α (a kind gift of DA. Compton), anti-TPX2 (Novus Biologicals), anti-Aurora A (BD Transduction Laboratories), anti-centrin 20H5 (a kind gift of JL Salisbury) and anti-pericentrin (Abcam) antibodies. Secondary antibodies were Alexa-488 anti-rabbit (Molecular Probes, Eugene, OR) and X-Red anti-mouse (Jackson Laboratories, Bar Harbor, ME) antibodies. DNA was counterstained in 0.05 mg/ml 4,6-diamidino-2-phenylindole (DAPI, Sigma, St Louis, MO) and slides were viewed under an Olympus AX70 microscope using a 100×/1.35NA objective. Images were acquired using a TCH-1.4ICE camera (Tucsen Photonics, China) controlled by ISCapture and processed using Photoshop CS software.

### Statistical analyses

Data are presented as the mean of at least three independent experiments along with standard error (SE). The One-way Analysis of Variance (ANOVA) and the Tukey–Kramer post hoc test were applied to compare data. Probability values (p) < 0.05 were considered statistically significant. Statistical analysis of data was performed using GraphPad software Instat version 3.02 (GraphPad Software, San Diego, CA).

### Computational studies of tubulin-ligand interactions

Docking simulations of 3,5,4′-TMS, 3,4,4′-TMS, combretastatin A4 and colchicine interaction with the γ/γ-tubulin or α/β-tubulin dimer were performed using the program AutoDock v. 4.2.6 [38]. The experimentally determined three-dimensional structure of the γ/γ–tubulin dimer (PDB ID: 3CB2, Resolution: 2.3 Å) [39] and the homology model built for the α/β-tubulin dimer, using as template the experimentally determined 3D structure from sheep (PDB ID: 5EYP; Resolution: 1.9 Å) [40], were used as protein targets. A detailed description of the steps preparatory to the docking procedure (i.e., receptor and ligand preparation and binding site prediction), as well as AutoDock parameters, are reported in Additional file 7: Additional Information.

## Additional files

**Additional file 1: Figure S1.** Representative flow cytometric histograms of HCT116 cells treated with 3,5,4′-TMS or 3,4,4′-TMS. X axis = DNA content, Y axis = number of events.

**Additional file 2: Figure S2.** Alterations of mitotic spindle structure, cell cycle progression and apoptosis in SW620 cells. (A) SW620 cells were treated with 80 µM 3,4,4′-TMS for 2 h, fixed and stained with anti-α-tubulin and anti-γ-tubulin antibodies. 3D projections of confocal images of untreated and treated cells are shown. Scale bar, 10 μm. (B) Alterations in cell cycle progression and apoptosis following treatment of SW620 cells with 3,5,4′-TMS. (C) Alterations in cell cycle progression and apoptosis following treatment of SW620 cells with 3,4,4′-TMS.

**Additional file 3: Figure S3.** Localization of different spindle pole proteins in 3,4,4′-TMS treated cells. HCT116 cells were exposed to 40 μM 3,4,4′-TMS and then immunostained with anti-Aurora A and anti-α-tubulin antibodies, anti-TPX2 and anti-α-tubulin antibodies or anti-Kif2a and anti-α-tubulin antibodies. DNA was counterstained by DAPI staining.

**Additional file 4: Figure S4.** Redocking of colchicine to the α/β-tubulin dimer. The α- and β-tubulin monomers are shown as ribbon and coloured light and dark green, respectively. Colchicine and GTP are shown as sticks and coloured by atom type: N, blue; O, red; P, orange; C, yellow for colchicine imported from α/β-tubulin crystal structure to the homology model, and light brown for colchicine positioned by docking.

**Additional file 5: Figure S5.** Comparison of colchicine binding to γ/γ- and α/β-tubulin dimer. β- and γ-tubulin are shown as ribbon and coloured dark green and cyan, respectively. Only β-tubulin and the structurally corresponding γ**-**tubulin monomers are shown, for clarity. Colchicine ligands are shown as sticks and coloured by atom type: N, blue; O, red; P, orange; C, yellow for colchicine imported from α/β-tubulin crystal structure to the homology model, and white for colchicine docked to γ-tubulin.

**Additional file 6: Figure S6.** Chemical structure of the intermediate and final products of the synthesis of 3,5,4′-TMS and 3,4,4′-TMS. 3,5,4′-TMS (5a) and 3,4,4′-TMS (5b) were synthetized by the classical synthesis of olefins using Wittig reaction with a slight modification. The ylide was generated by LiOH starting from the phosphonium salt (2). Then, the olefin products were obtained as mixture of cis and trans isomers by reaction with benzaldehydes 3a or 3b. The Z/E mixtures (4) were converted to the E-isomers 5a and 5b by heating with catalytic amounts of iodine in refluxing heptane.

**Additional file 7.** Additional materials and methods.

## Abbreviations

RSV: resveratrol
3: 5,4′-TMS, 3,5,4′-trimethoxystilbene
MT: microtubule
3: 4,4′-TMS, 3,4,4′-trimethoxystilbene
PBS: phosphate buffered saline
TPX2: targeting protein for Xklp2
Kif2a: kinesin family member 2A
Eg5: kinesin-related motor protein Eg5
