# Supplementary figures and images for: A novel resveratrol derivative induces mitotic arrest, centrosome fragmentation and cancer cell death by inhibiting γ-tubulin
Source: Cell Div. 2019 Apr 10;14:3. doi: 10.1186/s13008-019-0046-8 (PMC6457039; doi:10.1186/s13008-019-0046-8)

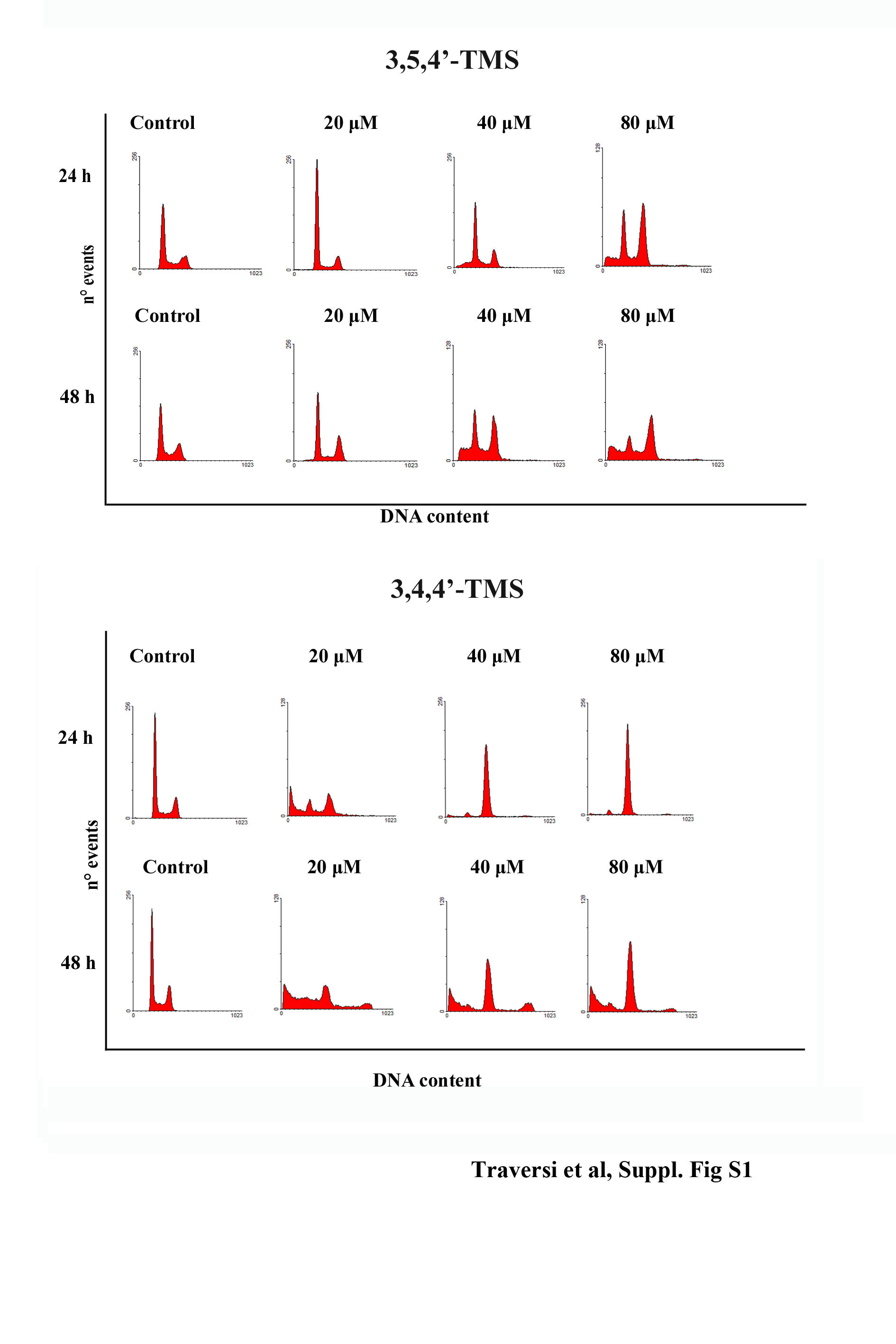

Supplement: Supplementary file 1 — Additional file 1: Figure S1. Representative flow cytometric histograms of HCT116 cells treated with 3,5,4′-TMS or 3,4,4′-TMS. X axis = DNA content, Y axis = number of events. [file 13008_2019_46_MOESM1_ESM.tif]

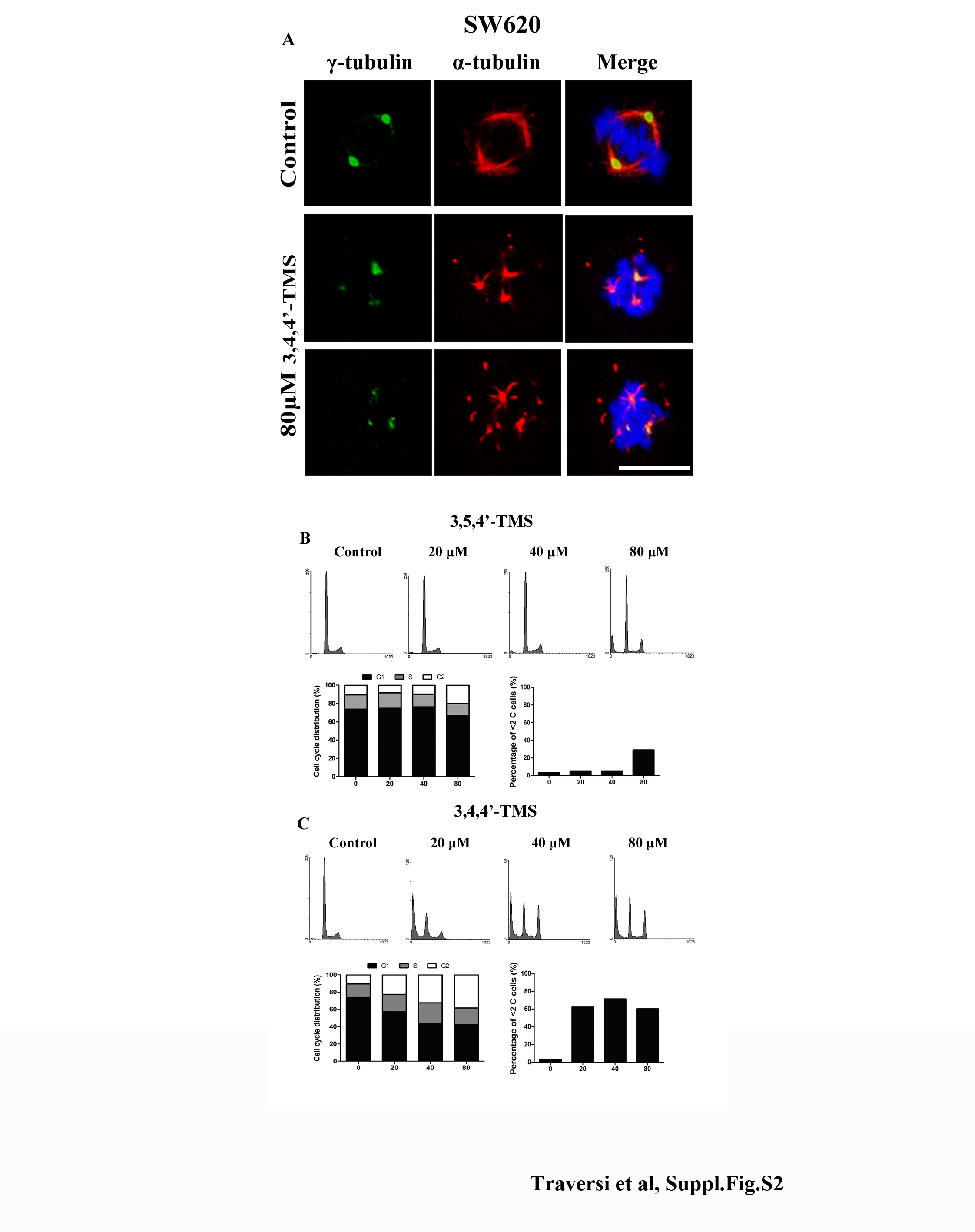

Supplement: Supplementary file 2 — Additional file 2: Figure S2. Alterations of mitotic spindle structure, cell cycle progression and apoptosis in SW620 cells. (A) SW620 cells were treated with 80 µM 3,4,4′-TMS for 2 h, fixed and stained with anti-α-tubulin and anti-γ-tubulin antibodies. 3D projections of confocal images of untreated and treated cells are shown. Scale bar, 10 μm. (B) Alterations in cell cycle progression and apoptosis following treatment of SW620 cells with 3,5,4′-TMS. (C) Alterations in cell cycle progression and apoptosis following treatment of SW620 cells with 3,4,4′-TMS. [file 13008_2019_46_MOESM2_ESM.tif]

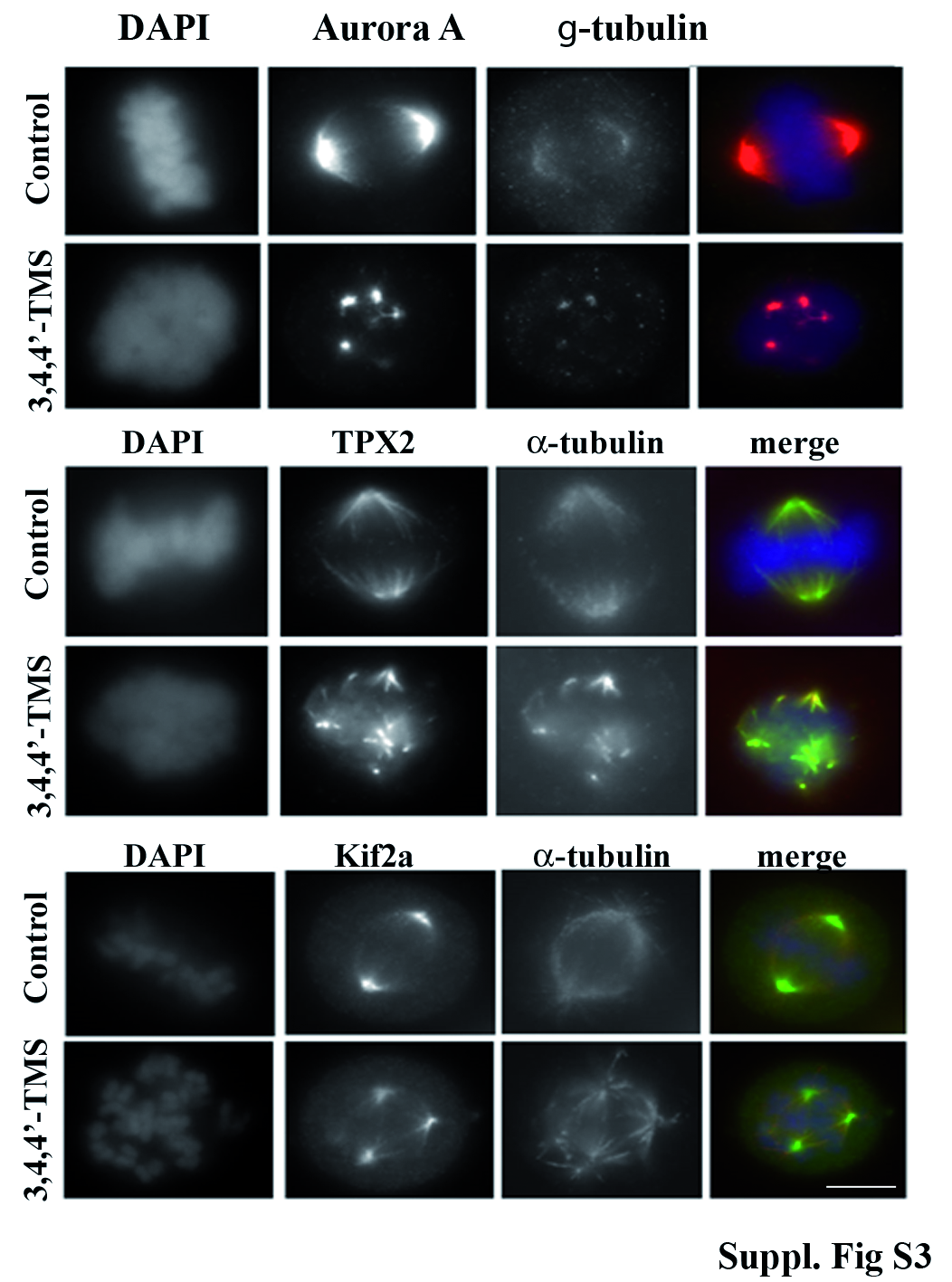

Supplement: Supplementary file 3 — Additional file 3: Figure S3. Localization of different spindle pole proteins in 3,4,4′-TMS treated cells. HCT116 cells were exposed to 40 μM 3,4,4′-TMS and then immunostained with anti-Aurora A and anti-α-tubulin antibodies, anti-TPX2 and anti-α-tubulin antibodies or anti-Kif2a and anti-α-tubulin antibodies. DNA was counterstained by DAPI staining. [file 13008_2019_46_MOESM3_ESM.tif]

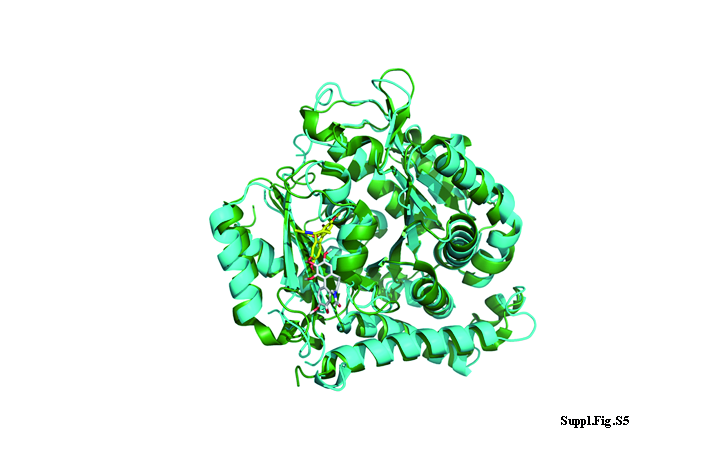

Supplement: Supplementary file 5 — Additional file 5: Figure S5. Comparison of colchicine binding to γ/γ- and α/β-tubulin dimer. β- and γ-tubulin are shown as ribbon and coloured dark green and cyan, respectively. Only β-tubulin and the structurally corresponding γ-tubulin monomers are shown, for clarity. Colchicine ligands are shown as sticks and coloured by atom type: N, blue; O, red; P, orange; C, yellow for colchicine imported from α/β-tubulin crystal structure to the homology model, and white for colchicine docked to γ-tubulin. [file 13008_2019_46_MOESM5_ESM.tif]

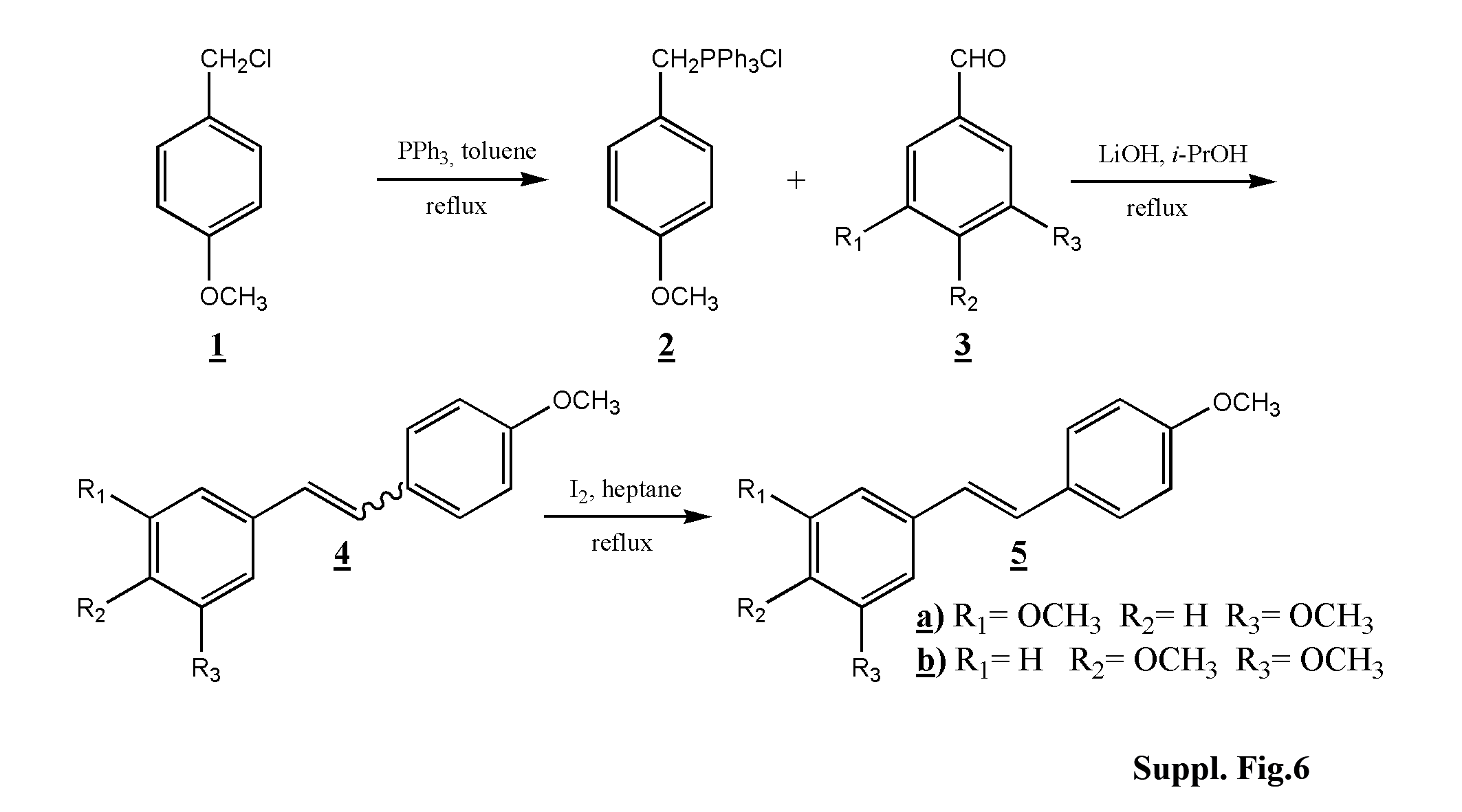

Supplement: Supplementary file 6 — Additional file 6: Figure S6. Chemical structure of the intermediate and final products of the synthesis of 3,5,4′-TMS and 3,4,4′-TMS. 3,5,4′-TMS (5a) and 3,4,4′-TMS (5b) were synthetized by the classical synthesis of olefins using Wittig reaction with a slight modification. The ylide was generated by LiOH starting from the phosphonium salt (2). Then, the olefin products were obtained as mixture of cis and trans isomers by reaction with benzaldehydes 3a or 3b. The Z/E mixtures (4) were converted to the E-isomers 5a and 5b by heating with catalytic amounts of iodine in refluxing heptane. [file 13008_2019_46_MOESM6_ESM.tif]
