# Supplementary material for: A novel resveratrol derivative induces mitotic arrest, centrosome fragmentation and cancer cell death by inhibiting γ-tubulin
Source: Cell Div. 2019 Apr 10;14:3. doi: 10.1186/s13008-019-0046-8 (PMC6457039; doi:10.1186/s13008-019-0046-8)
Supplement: Supplementary file 7 — Additional file 7. Additional materials and methods. [file 13008_2019_46_MOESM7_ESM.docx]

**Additional materials and methods**

**Synthesis of (E)-3,5,4’-TMS and (E)-3,4,4’-TMS**

**Experimental conditions**

The solvents (HPLC grade) and the reagents used were purchased from Sigma-Aldrich S.r.l. (Milan, Italy). Flash column chromatography was conducted on Silica gel 230-400 mesh (Merck S.p.A., Milan). Reactions were monitored by TLC using Merck silica gel 60F-254 plates with UV indicator and visualized with ultraviolet light, or iodine spray, or phosphomolybdic acid (10% sol in EtOH). HPLC analyses were performed by using an Agilent 1260 Liquid Chromatography equipped with a Diode Array Detector (DAD). The 1H and 13C spectra were recorded with a Varian Mercury 3000 at 300 MHz and 75 MHz, in CDCl3 as solvent. 1H-NMR chemical shifts (δ) are expressed in parts per million (ppm), coupling constants (J) were measured in Hertz (Hz), and coupling patterns are indicated as s (singlet), d (doublet), t (triplet), q (quartet), bs (broad singlet), bd (broad doublet), m (multiplet). 13C-NMR results (in ppm) are measured in correlation to CDCl3 (δ=77.0 ppm for centerline).

**Synthesis of 4-methoxy-benzyl-triphenyl-phosphonium chloride 2**

To a solution of 4-methoxybenzyl chloride 1 (30 mmol) in toluene (30 ml) was added a solution of triphenyl-phosphine (40 mmol) in toluene (30ml). After being heated at reflux about 5 hr, the reaction mixture was cooled at room temperature and a first crop of product was collected by filtration. The filtrate was then refluxed for additional 5 hr and second crop of product precipitated. The collected crops were crystallized from ethanol to give 4-methoxybenzyl-triphenyl-phosphonium chloride 2 in 95% yield. 1H-NMR (300 MHz, CDCl3): d (ppm) 7.80–7.50 (m, 15H), 6.99 (d, 2H, J = 8 Hz), 6.66 (d, 2H, J = 8 Hz), 5.30 (d, 2H, J = 12 Hz), 3.72 (s, 3H).

**Synthesis of stilbenes 5a and 5b**

Lithium hydroxide (16 mmol) was added to a stirred solution of the already described phosphonium salt (12 mmol) in isopropyl alcohol (40 mL). After 15 min a solution of benzaldehyde 3a or 3b (10 mmol) was added to the mixture and the reaction was carried out at reflux. The reaction was continued until complete consumption of the aldehyde (7 hr), monitored by TLC and HPLC, then quenched with water and extracted with ethyl acetate. The organic phase was washed with brine until neutral, dried with Na2SO4 and evaporated in vacuum. The residue was purified by flash chromatography (SiO2) eluting with hexane/Et2O 9:1 (v/v) to afford the desired product (10.2 mmol, 92%) as mixture of two isomers (E/Z - 44/56).

The mixtures of two stereoisomers E and Z 4 (10 mmol) were dissolved in heptane (30 mL). A catalytic amount of iodine (ca. 10-20 mg) was added to this solution and heated at reflux for 12 hours. The reaction was monitored by TLC and HPLC. When only the trans form was present, the reaction mixture was diluted with 30 mL of Et2O and washed with saturated aqueous NaHSO3 (3 x 20 mL) and after with NaCl (3 x 20 mL). The organic layer was dried over MgSO4 and concentrated in vacuum to provide the desired E-isomer 5a or 5b. The isolated compounds were characterized by 1H-NMR and 13C-NMR spectroscopy.

**5a.** White solid (8.15 mmol, 81% yield), mp 54-56 °C (lit. [1] 55-57 °C). Rf = 0.3 (hexanes–EtOAc, 9:1). 1H-NMR (300 MHz, CDCl3): δ (ppm) 7.43 (d, 2H, J = 8 Hz), 7.03 (d, 1H, J = 16 Hz), 6.88 (d, 2H, J = 8 Hz), 6.87 (d, 1H, J = 16 Hz), 6.64 (d, 2H, J = 3 Hz), 6.37 (t, 1H, J = 3 Hz), 3.81 (s, 9H). 13C-NMR (75 MHz, CDCl3): δ (ppm) 161.0, 159.5, 148.5, 139.6, 129.9, 127.8(2×C), 120.0, 114.1(2×C), 111.1, 104.4, 99.7 55.9(OMe), 55.8 (OMe), 55.3 (OMe).

**5b.** White solid; (8.72 mmol, 87%); mp 137.1-138.9 °C (lit. [2] 136-137 °C); Rf = 0.3 (hexanes–EtOAc, 8:2). 1H NMR (400 MHz, CDCl3): δ (ppm) 7.43 (d, 2H, J=8.8 Hz, H-2’, H-6’), 7.05 (d, 1H, J=1.71 Hz, H-5), 7.02 (dd, 1H, J=1.95, 8.29 Hz, H-β), 6.81-6.94 (m, 5H, H-2, H-6, H-3’, H-5’, H-α), 3.94 (s, 3H, OMe), 3.90 (s, 3H, OMe), 3.82 (s, 3H, OMe); 13C NMR (100 MHz, CDCl3): δ (ppm) 159.0, 149.0, 148.5, 130.7, 130.3, 127.4(2×C), 126.3(2×C), 119.5, 114.1(2×C), 111.0, 101.4, 55.9 (OMe), 55.8 (OMe), 55.3 (OMe).

References

1) - Wang, B.J.; Zou, J.H.; Li, W.X.; Wang, Z.; Xu, B.; Li, S.; Zhai, Y.S.; Zhu, D.L.; Li, Q.Y.; Yang, G.W. J. Organomet. Chem. 2014, 749, 428-432.

2) - McNulty, J.; Das, P.; McLeod, D. Chem.-Eur. J. 2010, 16, 6756-6760.

**Computational studies of tubulin-ligand interactions**

**Receptor preparation**

The three-dimensional (3D) structure of γ/γ- and α/β-tubulin dimers have been experimentally determined by X-ray crystallography and are available from the Protein Data Bank (PDB: https://www.rcsb.org/) [Berman et al, 2000] with identifiers (ID) 3CB2 (Resolution: 2.3 Å) [Rice et al, 2008] and 5IJ9 (Resolution: 3.7 Å) [Ti et al., 2016], respectively. Given the low resolution of the latter structure, we built a molecular model of human α/β-tubulin dimer using as a template the 3D structure of sheep α/β-tubulin dimer in complex with colchicine, which has been experimentally determined by X-ray crystallography with high resolution (PDB ID: 5EYP; Resolution: 1.9 Å) [Ahmad et al, 2016].

We used the Blast program [Altschul et al, 1990] to search the NCBI protein database for proteins of known 3D structure homologous to human α- and β-tubulins. The best hits were the sequences of α- and β-tubulin from sheep, which have 100% and 91% sequence identity over 451 and 450 residues, respectively, with the human counterparts, and whose dimeric structure in complex with colchicine has been determined by X-ray crystallography with high resolution (PDB ID: 5EYP, Resolution: 1.9 Å) [Ahmad et al, 2016]. Importantly, β-tubulin is particularly conserved in the colchicine binding site region. According to the PDBSum server [Laskowski et al, 2018], the only two mutated residues in contact with colchicine (i.e., having at least one atom at a distance lower than 4.0 Å from a colchicine atom) in the sheep structure are Cys241 and Ile318. Their replacement by Ser and Val, respectively, in the human model, is not expected to involve a conformational change, given the isostery between Cys and Ser and the overlapping between Val and Ile for all the length of Val side-chain. For all of the above, the 3D structure of sheep α/β-tubulin dimer is a suitable template to build an accurate model of the human homologue.

The 3D atomic model of human α/β-tubulin dimer was built using the program Chimera [Pettersen et al, 2004] to computationally mutate the residues of sheep β-tubulin that are not conserved in human. Because of the high resolution of the template and high sequence identity between model and template, this model is expected to provide a more accurate representation of human α/β-tubulin than the low resolution X-ray structure. Further, since the human and sheep structures have been determined in the absence and presence of colchicine, respectively, the latter is more likely to represent the conformation assumed in the presence of ligands bound to the colchicine binding site, where RSV analogues have been previously predicted to bind [Mazué et al, 2010].

Prior to docking studies, crystallographic waters were removed from the structure of γ/γ-tubulin. AutoDock Tools (ADT) 1.5.6 was used to add hydrogens, automatically assign Gasteiger charges, and merge non-polar hydrogens to both γ/γ-tubulin structure and α/β-tubulin model.

**Ligand preparation**

The structure of 3,4,4'-TMS is not available from Ambinter (http://www.ambinter.com/), ZINC [Sterling & Irwin, 2015] or other standard databases, and was built using the program Bioclipse v. 2.6.2 [Spjuth et al, 2009]. The structures of all other compounds were downloaded from the Ambinter database (3,5,4'-TMS: Amb8394413; combretastatin A-4: Amb8396465; colchicine: Amb19930400).

All compounds were converted in mol2 format using Open Babel v. 2.3.1 [O’Boyle et al, 2011]. The molecular editor and visualizer Avogadro (v. 1.2.0) [Hanwell et al, 2012] was used to add hydrogen atoms and optimize all the structures using Universal Force Field [Rappe et al, 1992]. ADT was used to assign Gasteiger charges, merge non-polar hydrogens, set active torsions and determine planar cyclic carbons.

**Binding site prediction**

To identify the γ/γ-tubulin regions most likely to bind the aforementioned ligands we used three distinct approaches.

1) Comparative approach. We obtained the list of tubulins of known structure from the Pfam database (https://pfam.xfam.org/) [Finn et al, 2016], and downloaded the corresponding co-ordinate files from the PDB. Then we used the Combinatorial Extension (CE) program [Shindyalov & Bourne, 1998] to align all tubulin monomers to one another, and uploaded the resulting multiple structure alignment in PyMol v. 1.8.2.1 (The PyMOL Molecular Graphics System, Version 1.8, Schrödinger, LLC) to visualize the results and select the structures that had been determined in complex with organic molecules different from GDP/GTP. The residues in contact with these molecules were identified using the PDBsum webserver [Laskowski et al, 2018].

2) Geometric approach. The AutoLigand [Harris et al, 2007] module of the ADT package was used to identify ligand binding sites in γ/γ-tubulin structure and α/β-tubulin model. For both proteins, a grid search space of 1.0 Å was set to enclose the entire protein, and a fill size of 200 fill points was used. For the grid box the following parameters were adopted: center on 26.291, 24.308 and -16.613, with dimension 68x66x88 for γ/γ-tubulin.

3) Blind docking simulations. A specific docking site was not specified; therefore, the ligand was allowed to interact with the whole protein surface.

**Docking simulations**

The affinity grid box was calculated using the Autogrid program v.4.2.6 to include the main cavity found by Autoligand. AutoDock parameters were: spacing value at 0.375 Å, center on coordinates 26.291, 24.308 and -16.613, and with 70x90x60 grid points for γ/γ-tubulin; center on 23.798, 13.388 and -18.158, with 46x52x44 grid points for α/β-tubulin.

Molecular docking was performed using the Lamarkian genetic algorithm implemented in AutoDock. Docking parameters were as follows: 100 Genetic Algorithm (GA) runs, population size of 150, RMS cluster tolerance of 1.5 Å. All other parameters were left as default.

The docking procedure was validated by colchicine “redocking” to α/β-tubulin dimer (See Supplementary Figure 3).

References

Ahmad S, Pecqueur L, Dreier B, Hamdane D, Aumont-Nicaise M, Pluckthun A, Knossow M, Gigant B. Destabilizing an interacting motif strengthens the association of a designed ankyrin repeat protein with tubulin. Sci Rep (2016) 6: 28922-28922.

Altschul SF, Gish W, Miller W, Myers EW, Lipman DJ. Basic local alignment search tool. J. Mol. Biol. (1990) 215: 403-410.

Berman HM, Westbrook J, Feng Z, Gilliland G, Bhat TN, Weissig H, Shindyalov IN, Bourne PE. The Protein Data Bank. Nucleic Acids Research (2000) 28: 235-242.

Finn RD, Coggill P, Eberhardt RY, Eddy SR, Mistry J, Mitchell AL, Potter SC, Punta M, Qureshi M, Sangrador-Vegas A, Salazar GA, Tate J, Bateman A. The Pfam protein families database: towards a more sustainable future. Nucleic Acids Research (2016) Database Issue 44: D279-D285.

Friesen DE, Barakat KH, Semenchenko V, Perez-Pineiro R, Fenske BW, Mane J, Wishart DS, Tuszynski JA. Discovery of small molecule inhibitors that interact with γ-tubulin. Chem Biol Drug Des (2012) 79: 639-652.

Hanwell MD, Curtis DE, Lonie DC, Vandermeersch T, Zurek E, Hutchison GR. Avogadro: An advanced semantic chemical editor, visualization, and analysis platform. Journal of Cheminformatics (2012) 4: 17.

Harris R, Olson AJ, Goodsell DS. Automated prediction of ligand-binding sites in proteins. Proteins: Structure, Function and Bioinformatics (2007) 70: 1506-1517.

Laskowski RA, Jabłońska J, Pravda L, Vařeková RS, Thornton JM. PDBsum: Structural summaries of PDB entries. Prot. Sci. (2018) 27: 129-134.

O'Boyle NM, Banck M, James CA, Morley C, Vandermeersch T, Hutchison GR. Open Babel: An open chemical toolbox. Journal of Cheminformatics (2011) 3: 33.

Pettersen EF, Goddard TD, Huang CC, Couch GS, Greenblatt DM, Meng EC, Ferrin TE. UCSF Chimera--a visualization system for exploratory research and analysis. J Comput Chem (2004) 25: 1605-1612.

Rappe AK, Casewit CJ, Colwell KS, Goddard WA III, Skiff WM. UFF, a Full Periodic Table Force Field for Molecular Mechanics and Molecular Dynamics Simulations. J. Am. Chem. Soc. (1992) 114: 10024–10035.

Shindyalov IN, Bourne PE. Protein structure alignment by incremental combinatorial extension (CE) of the optimal path. Protein Engineering (1998) 11: 739-747.

Sievers F, Wilm A, Dineen D, Gibson TJ, Karplus K, Li W, Lopez R, McWilliam H, Remmert M, Söding J, Thompson JD, Higgins DG. Fast, scalable generation of high-quality protein multiple sequence alignments using Clustal Omega. Mol. Syst. Biol. (2011) 7: 539.

Spjuth O, Alvarsson J, Berg A, Eklund M, Kuhn S, Mäsak C, Torrance G, Wagener J, Willighagen EL, Steinbeck C, Wikberg JES. Bioclipse 2: A scriptable integration platform for the life sciences. BMC Bioinformatics (2009) 5: 397.

Sterling & Irwin. ZINC 15 – Ligand Discovery for Everyone J. Chem. Inf. Model (2015) 55: 2324–2337.

The UniProt Consortium. UniProt: the universal protein knowledgebase. Nucleic Acids Research (2017) 45: D158-D169.

Ti SC, Pamula M.C., Howes S.C., Duellberg C., Cade N.I., Kleiner R.E., Forth S., Surrey T, Nogales E, Kapoor TM. Mutations in Human Tubulin Proximal to the Kinesin-Binding Site Alter Dynamic Instability at Microtubule Plus- and Minus-Ends. Dev. Cell (2016) 37: 72-84.

Waight AB, Bargsten K, Doronina S, Steinmetz MO, Sussman D, Prota AE. Structural Basis of Microtubule Destabilization by Potent Auristatin Anti-Mitotics. Plos One (2016) 11: e0160890-e0160890.
